# Breast cancer stem cell-like cells generated during TGFβ-induced EMT are radioresistant

**DOI:** 10.18632/oncotarget.25240

**Published:** 2018-05-04

**Authors:** Julie Konge, François Leteurtre, Maud Goislard, Denis Biard, Sandrine Morel-Altmeyer, Aurélie Vaurijoux, Gaetan Gruel, Sylvie Chevillard, Jérôme Lebeau

**Affiliations:** ^1^ CEA, Institut de Biologie François Jacob, DSV, iRCM, SREIT, Laboratoire de Cancérologie Expérimentale (LCE), Université Paris-Saclay, F-92265 Fontenay-aux-Roses, France; ^2^ CEA, Institut de Biologie François Jacob, SEPIA, Team Cellular Engineering and Human Syndromes, Université Paris-Saclay, F-92265 Fontenay-aux-Roses, France; ^3^ Institut de Radioprotection et de Sureté Nucléaire (IRSN), Laboratoire de Dosimétrie Biologique, 92262 Fontenay-aux-Roses Cedex, France

**Keywords:** epithelial-mesenchymal transition, breast cancer, radioresistance, cancer stem cells

## Abstract

Failure of conventional antitumor therapy is commonly associated with cancer stem cells (CSCs), which are often defined as inherently resistant to radiation and chemotherapeutic agents. However, controversy about the mechanisms involved in the radiation response remains and the inherent intrinsic radioresistance of CSCs has also been questioned. These discrepancies observed in the literature are strongly associated with the cell models used. In order to clarify these contradictory observations, we studied the radiosensitivity of breast CSCs using purified CD24^−/low^/CD44^+^ CSCs and their corresponding CD24^+^/CD44^low^ non-stem cells. These cells were generated after induction of the epithelial-mesenchymal transition (EMT) by transforming growth factor β (TGFβ) in immortalized human mammary epithelial cells (HMLE). Consequently, these 2 cellular subpopulations have an identical genetic background, their differences being related exclusively to TGFβ-induced cell reprogramming. We showed that mesenchymal CD24^−/low^/CD44^+^ CSCs are more resistant to radiation compared with CD24^+^/CD44^low^ parental cells. Cell cycle distribution and free radical scavengers, but not DNA repair efficiency, appeared to be intrinsic determinants of cellular radiosensitivity. Finally, for the first time, we showed that reduced radiation-induced activation of the death receptor pathways (FasL, TRAIL and TNF-α) at the transcriptional level was a key causal event in the radioresistance of CD24^−/low^/^CD44+^ cells acquired during EMT.

## INTRODUCTION

The epithelial-to-mesenchymal transition (EMT) program is critical for tumor progression and acquisition of invasive potential. A growing body of evidence has demonstrated a direct link between EMT progression and the emergence of cancer stem cells (CSCs), suggesting that the EMT program plays critical role in the generation and maintenance of CSCs or CSC-like cells. In breast cancers, tumor cells undergoing EMT gain CSC properties, including the ability to self-renew and tumorigenicity, and exhibit a CD24^−/low^/CD44^+^ phenotype [1, 2]. Although the relevance of the CSC model and the existence of CSCs in solid tumors are frequently debated, this model describes more accurately than a stochastic model why many epithelial tumor cells first respond to conventional treatments and then recur [3]. Failure of conventional treatments has commonly been associated with CSCs survival [4–7]. While resistance to radiation therapy has been reported to be a defining characteristic of CSCs from various tumor types, including breast tumors, different mechanisms may underline resistance of CSCs. The main mechanisms proposed for the genotoxic stress survival of CSCs could be underpinned by one or more of the following properties: - i) efficient activation of DNA damage checkpoints combined with high DNA repair activity, - ii) low level of endogenous reactive oxygen species (ROS) associated with increased expression of free radical scavenging systems, - iii) cell cycle distribution and intracellular level of activated checkpoint proteins. Depending on the studies, these mechanisms may be exclusive or not [4, 6, 8–10]. Nevertheless, the inherent intrinsic radioresistance of CSCs has been challenged and some studies claim that CSCs are not more radioresistant, or even more sensitive to radiation than non-stem cells [11–14]. The origin of these discrepancies could be related to the different cell models used, especially when properties of different cell lines were compared or when stem-like tumor cells were obtained after forced expression of transcription factors (TFs) involved in the EMT/CSC phenotype. In breast cancer cells, ectopic expression of one EMT-TF or of H-RAS^G12V^ endows mesenchymal CD24^low^/CD44^high^ cells with stem-like properties [1, 2]. However, the capacities of these cells to resume all the properties of “purified” mesenchymal CD24^low^/CD44^high^ cells remain elusive. In addition, the confusion is reinforced when several mechanisms are proposed in the literature to explain the radiosensitivity observed in different studies. Finally, when the data for predictive value of CSC markers in the setting of radiation therapy of breast cancer were meta-analyzed, the results obtained were confusing, even for CSC CD24^low^/CD44^high^ labeling. The main conclusions were an obvious lack of data and a need for more precisely defined endpoints to evaluate the radiation response of CSCs [15].

In order to improve our knowledge of the intrinsic radiosensitivity of CSCs generated during EMT in breast tumors, we chose to work with purified CD24^+^/CD44^low^ and CD24^−/low^/CD44^+^ cells obtained after induction of EMT by transforming growth factor β (TGFβ) in immortalized human mammary epithelial cells (HMLE). TGFβ is a major driving force of the EMT genetic program and activation of TGFβ signaling pathways is one of the key biological processes during tumor progression and initiation of metastasis [16, 17]. TGFβ-driven EMT is sufficient to generate migrating CSCs by directly linking the acquisition of cellular motility with the maintenance of tumor-initiating “stemness” capacity. The cell model has been developed and used by A. Weinberg's team to study the role of EMT in CSC generation and the underlying mechanisms [1]. The main advantage of that model is that it can be used to compare CD24^+^/CD44^low^ and CD24^−/low^/CD44^+^ cells obtained upon induction of EMT in the same genetic background.

Using that cell model, in which the appearance of EMT and CD24^−/low^/CD44^+^ cells is exclusively linked to TGFβ-induced cell reprogramming, we compared the radiosensitivity of CSC-like cells and non-stem cells. We showed that mesenchymal CD24^−/low^/CD44^+^ cells generated during TGFβ-induced EMT are less sensitive to ionizing radiation than parental epithelial CD24^+^/CD44^low^ cells. This radioresistance was associated with cell cycle redistribution and with a reduced proportion of polyploid cells after irradiation, both of which lead to avoidance of mitotic catastrophe and subsequently to a higher cloning efficiency. Low ROS concentration and elevated antioxidant activity were also observed in CD24^−/low^/CD44^+^ cells. Finally, late cell death observed after irradiation was related to the activation of extrinsic death receptor pathways, and the strongly reduced level of activation in CD24^−/low^/CD44^+^ cells appeared to be a causal key event in the radioresistance of these cells. Overall, our data reveal that radioresistance is an intrinsically acquired characteristic of mesenchymal CD24^−/low^/CD44^+^ cells generated during TGFβ-induced EMT, and that more efficient ROS scavenging in these cells may impede activation of death receptor pathways following irradiation.

## RESULTS

### CD24^−/low^/CD44^+^ cells obtained upon a TGFβ-induced EMT have acquired resistance to radiation-induced apoptosis

**Figure 1:**
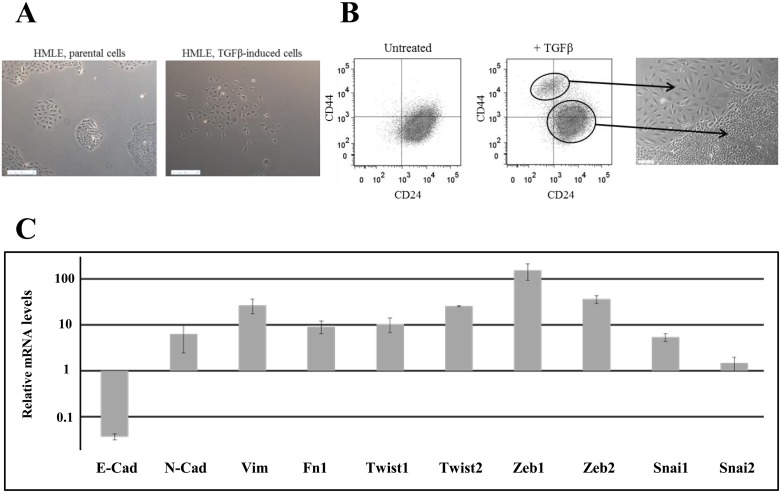
Characterization of TGFβ-induced CD24^−/low^/CD44^+^ cells (**A**) Phase-contrast image of HMLE parental cells (left) and HMLE TGFβ-induced cells (right). (**B**) Flow cytometry characterization of HMLE parental and TGFβ-induced cells. Parental cells display essentially CD24^+^/CD44^low^ labeling, and TGFβ treatment led to the appearance of a CD24^−/low^/CD44^+^ cell population. CD24^+^/CD44^low^ cells were morphologically cuboidal-like epithelial cells, and CD24^−/low^/CD44^+^ cells were fibroblast-like, mesenchymal cells. (**C**) Analysis by qRT-PCR of the relative expression of the mRNAs encoding E-cadherin, N-cadherin, vimentin, fibronectin 1, Twist1, Twist2, Zeb1, Zeb2, Snail1 and Snail2. Normalization was performed as indicated in Materials and Methods. For each gene, expression in CD24^+^/CD44^low^ cells was normalized to 1 and the ratio of relative mRNA level of CD24^−/low^/CD44^+^ cells to CD24^+^/CD44^low^ cells was presented. Each value corresponds to the mean value of at least 2 independent PCRs performed from 3 independent experiments. Error bars correspond to SEM.

EMT was induced in HMLE cells by prolonged exposure (5–12 days) to TGFβ1. We observed the appearance of 5–15% of morphologically fibroblast-like, mesenchymal cells, which were significantly different from the cuboidal-like epithelial phenotype of the parental cells (Figure 1A). Parental cells were characterized essentially by CD24^+^/CD44^low^ labeling in fluorescence-activated cell sorting (FACS) analysis, and, as anticipated, after TGFβ1 treatment, we detected a CD24^−/low^/CD44^+^ cell population (Figure 1B and Supplementary Figure 1), indicating that EMT generates stem cell-like cells. The 2 populations were isolated by FACS and grown separately in order to study and compare their properties. EMT was confirmed in stem-like CD24^−/low^/CD44^+^ cells through the upregulation of mRNAs encoding the main transcription factors driving EMT (Twist, Snail, Zeb …), the downregulation of epithelial marker (E-cadherin) and the upregulation of mesenchymal markers (N-cadherin, vimentin, fibronectin) (Figure 1C).

**Figure 2:**
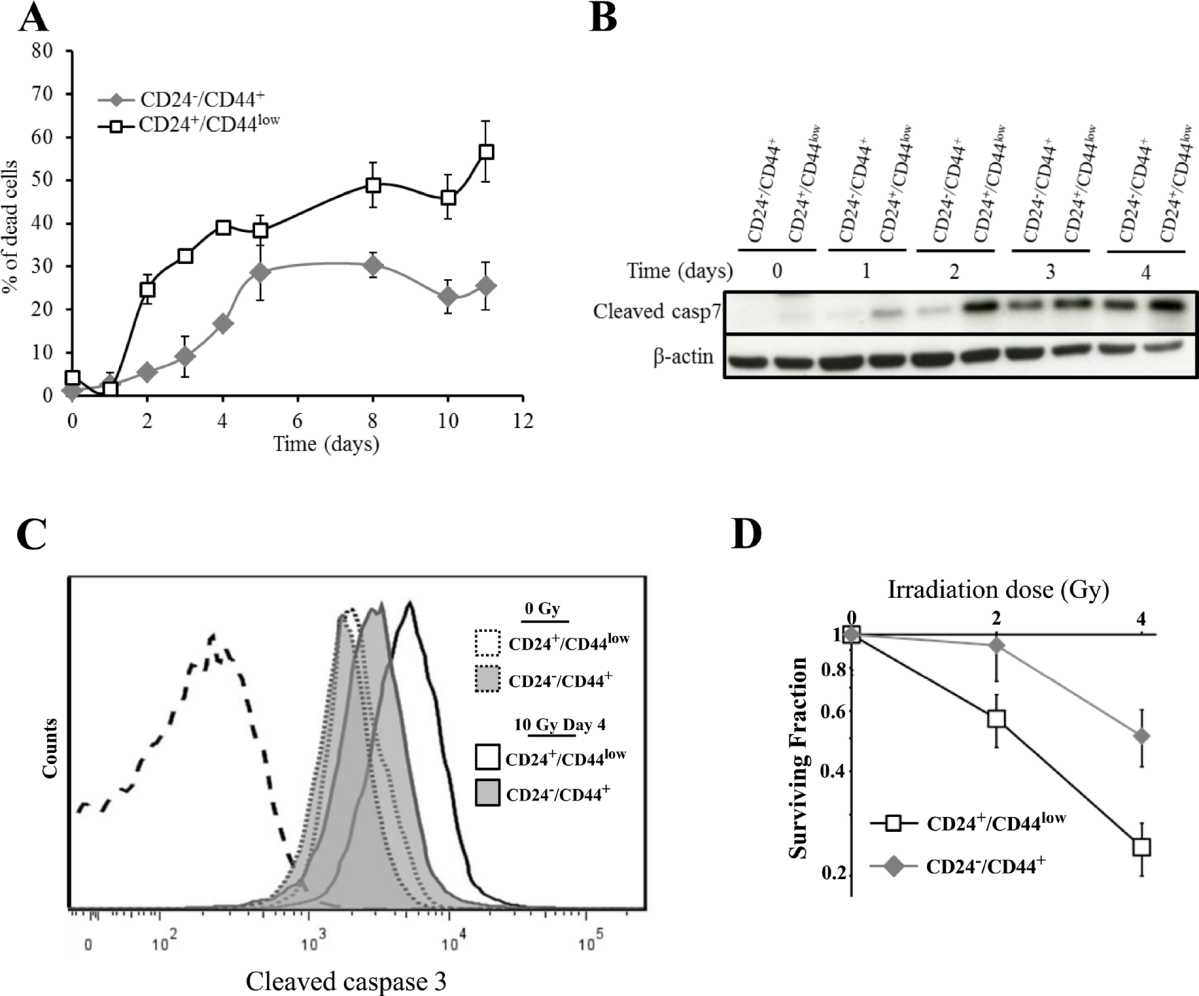
Analysis of cell death of CD24^+^/CD44^low^ cells and CD24^−/low^/CD44^+^ cells after 10 Gy irradiation (**A**) Time course of cell death of 10 Gy-irradiated CD24^+^/CD44^low^ cells and CD24^−/low^/CD44^+^ cells. Results correspond to the mean ± SD of 2 to 4 independent experiments. (**B**) Kinetics of cleavage of caspase 7 at different times after 10 Gy irradiation. The Western blot shown is representative of 3 independent experiments. (**C**) Flow cytometry characterization of cleaved caspase 3 before and 4 days after irradiation. Dotted line corresponds to isotypic control. Similar results were obtained in 3 different experiments. (**D**) Clonogenic cell survival curves for CD24^+^/CD44^low^ cells and CD24^−/low^/CD44^+^ cells exposed to 2 and 4 Gy irradiation. Error bars represent standard error from the mean for 3 separate experiments.

To investigate the radiosensitivity of parental and purified EMT-generated stem-like HMLE cells, the 2 cell populations were irradiated at 10 Gy (^137^Cs source) and monitored for 10 days. As in our group's previous reports using human mammary cell lines [18, 19], no early cell death was observed in either cell population, reflecting the lack of primary apoptotic response described in breast tumors. Interestingly, differential delayed cell death was observed between the 2 HMLE cell cultures (Figure 2A). In CD24^+^/CD44^low^ cells, cell death started on day 2 after γ-irradiation and increased to a plateau on day 5, and remained high for at least 11 days. In CD24^−/low^/CD44^+^ cells, cell death was significantly delayed and remained lower than in parental cells. As expected, in both cell cultures, delayed cell death was essentially linked to an apoptotic process: clear chromatin condensation and apoptotic bodies were detected in irradiated cells (data not shown), and cleavage of caspases 3 and 7, a hallmark of apoptosis, was observed. As for cell death, caspase 7 cleavage started earlier in CD24^+^/CD44^low^ cells and remained higher than in CD24^−/low^/CD44^+^ cells (Figure 2B). In the same way, FACS analysis of cleaved caspase 3 indicated that, 4 days after irradiation, the quantity of cleaved protein was significantly higher in CD24^+^/CD44^low^ than in CD24^−/low^/CD44^+^ cells (Figure 2C and Supplementary Figure 2). Again, radiation-induced apoptosis was initiated earlier and sustained at higher levels in CD24^+^/CD44^low^ than in CD24^−/low^/CD44^+^ cells.

We next investigated whether differences in the initiation timing of apoptosis and/or the lower level of cell death between the 2 cell cultures has an impact on long-term survival, as measured by the clonogenic cell survival assay after irradiation. For 2 and 4 Gy irradiation doses, the surviving fractions were significantly higher in CD24^−/low^/CD44^+^ than in CD24^+^/CD44^low^ cells (Figure 2D), indicating that CD24^−/low^/CD44^+^ cells generate a larger progeny than CD24^+^/CD44^low^ cells after irradiation.

Altogether, these results indicated that mesenchymal CD24^−/low^/CD44^+^ cells generated during TGFβ-induced EMT were less sensitive to ionizing radiation than parental epithelial CD24^+^/CD44^low^ cells.

### Avoidance of mitotic catastrophe but not DNA repair capacity contributes to differential radiosensitivity

**Figure 3:**
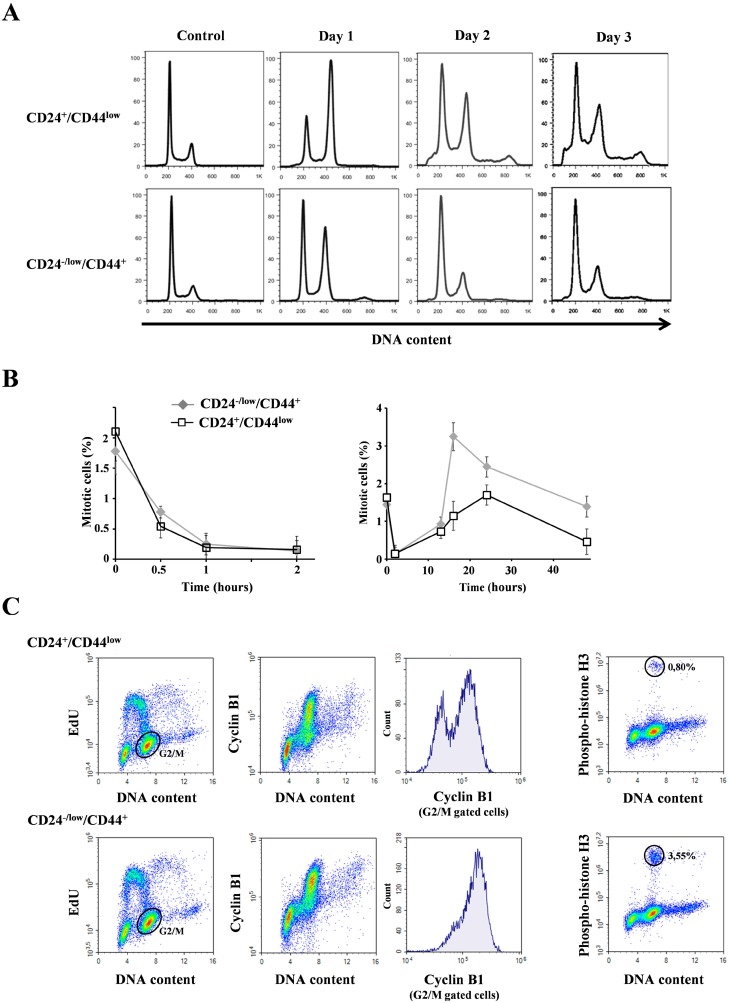
Analysis of cell cycle distribution in CD24^+^/CD44^low^ cells and CD24^−/low^/CD44^+^ cells after 10 Gy irradiation (**A**) Cell cycle analysis of 10 Gy-irradiated cells over 3 days. Similar results were obtained in at least 3 different experiments. (**B**) Mitotic index using phospho-histone H3 labelling at different times after 10 Gy irradiation: left 0–2 hours and right 0–48 hours after irradiation. Mean and SD of 4 independent experiments are presented. (**C**) Cyclin B1 and EdU labellings 16 hours after irradiation. Histograms of cyclin B1 were obtained from G2M cells (gated in the EdU/DNA content panel). Similar results were obtained in 2 different experiments. On the right, from the same sample, mitosis characterization using phospho-histone H3 labelling.

Delayed cell death may be accounted for by a mitotic catastrophe subsequent to prolonged radiation-induced growth arrest [19]. We investigated whether cell cycle distribution might play a role in the differential radiosensitivity of both cell populations. One day after 10 Gy irradiation, before cell death initiation, we observed a G2/M arrest (a 4n population) (Figure 3A) in agreement with the non-functional p53 status of HMLE cells. Two and 3 days after irradiation, the G2/M blockade was followed by the appearance of polyploid cells (8n population), essentially in CD24^+^/CD44^low^ cells, while the proportion of polyploid cells remained low in CD24^−/low^/CD44^+^cells. Concomitantly, cell death - as visualized by a sub-G1 population - started to occur, mainly in CD24^+^/CD44^low^ cells. To follow cells in M phase, further cell cycle analyses were performed using phospho-histone H3 labelling. Indeed, checkpoint activation upon irradiation led to a quick blockade of the transition from G2 phase to mitosis. This effect, occurred during the first hour post irradiation, was observed at all tested radiation doses 2, 4 and 10 Gy and was similar in CD24^−/low^/CD44^+^ and CD24^+^/CD44^low^ cells (Figure 3B–left and Supplementary Figure 3). Checkpoint activation is therefore efficient in both CD24^−/low^/CD44^+^ and CD24^+^/CD44^low^ cells.

Checkpoint exit and cell cycle recovery were then evaluated. Mitosis reappearance was observed 13 hours after a 10 Gy irradiation (Figure 3B-right and 3C-right). However, more mitoses were observed in CD24^−/low^/CD44^+^ than in CD24^+^/CD44^low^ cells, an effect obvious from 16 to 48 hours post-irradiation. Therefore, checkpoint inactivation, as stressed by mitosis reappearance, mainly occurred during the same time window in CD24^−/low^/CD44^+^ and CD24^+^/CD44^low^ cells.

The low percentage of mitosis observed in CD24^+^/CD44^low^ cells did correlate with the beginning of ploidy rise (ploidy reached 30% after 3 days, as measured by ploidy above 4n, that was an underestimation since G1 polyploid cells are not taking in account); this suggests that DNA endoreplication rather than mitosis entry could occur. Mitosis initiation requires activation of CDK1-cyclin B complexes, while endoreplication involves low CDK1-cyclin B activity (and activation of G1 phase CDKs). To distinguish between G2 cells driven toward M phase from those undergoing polyploidization, we performed cyclin B1 and EdU labellings (Figure 3C). CD24^+^/CD44^low^ cells in G2/M phase displayed lower cyclin B1 levels than CD24^−/low^/CD44^+^ cells. These results could therefore explain the high frequency of CD24^+^/CD44^low^ cells initiating endoreplication and polyploidyzation.

These results are fully in agreement with the definition of mitotic catastrophe, since irradiation has promoted loss of proliferative capacity, and cell death appeared after strong G2/M blockade and took place in cells during mitosis or after a failed mitosis in polyploid cells. Together, these observations demonstrate that CD24^−/low^/CD44^+^ cells, by displaying a lower proportion of polyploid cells, are able to avoid mitotic catastrophe.

**Figure 4:**
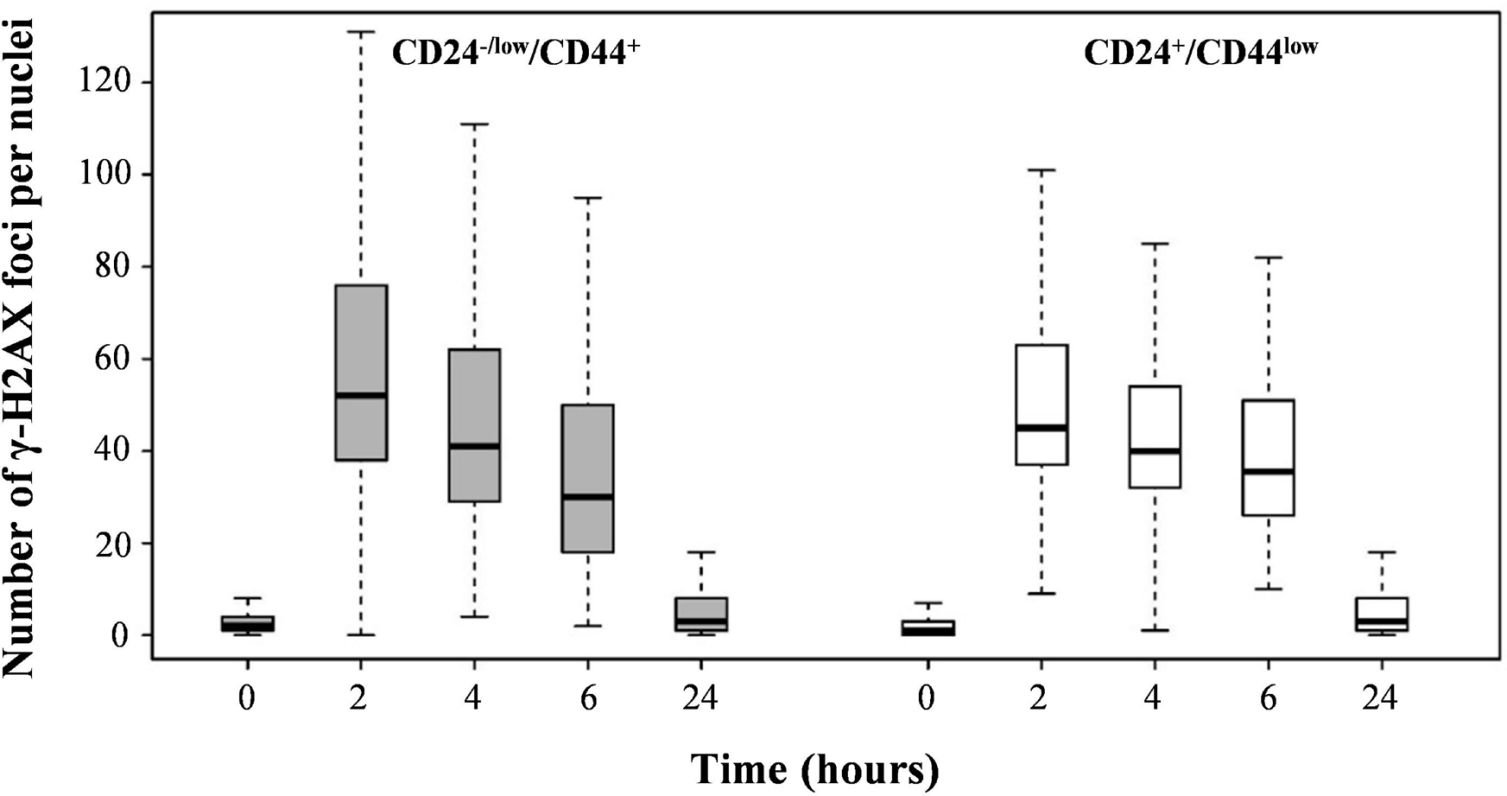
Analysis of global DSB repair in CD24^+^/CD44^low^ cells and CD24^−/low^/CD44^+^ cells after 4 Gy irradiation Number of γ-H2AX foci as a function of time upon 4 Gy irradiation.

We next investigated DNA double-strand break (DSB) repair capacity as a determining factor for differential radiosensitivity in CD24^+^/CD44^low^ *versus* CD24^−/low^/CD44^+^ cells. We quantified γ-H2AX foci as a DSB marker: their disappearance allows for overall monitoring of DNA repair. The kinetics of γ-H2AX foci disappearance after 4 Gy irradiation did not reveal significant differences between the 2 cell cultures (Figure 4). Thus, unexpectedly, the efficiency of DNA repair appears to be similar in CD24^−/low^/CD44^+^ and CD24^+^/CD44^low^ cells.

Taken together, these results indicate that TGFβ-induced EMT strongly modifies the cell cycle distribution after irradiation, while it does not significantly affect DNA repair capacity. Thus, the radiosensitivity of CD24^+^/CD44^low^ cells seems to be associated with a higher level of polyploid cells unable to sustain cell division in clonogenic assays. CD24^−/low^/CD44^+^radioresistance emanates from a probably more efficient G2/M blockade, which prevents the rise of the polyploid cell population and prevents a mitotic catastrophe.

### Radioresistance of CD24^−/low^/CD44^+^ cells is related to decreased intracellular ROS concentration and elevated antioxidant activity

**Figure 5:**
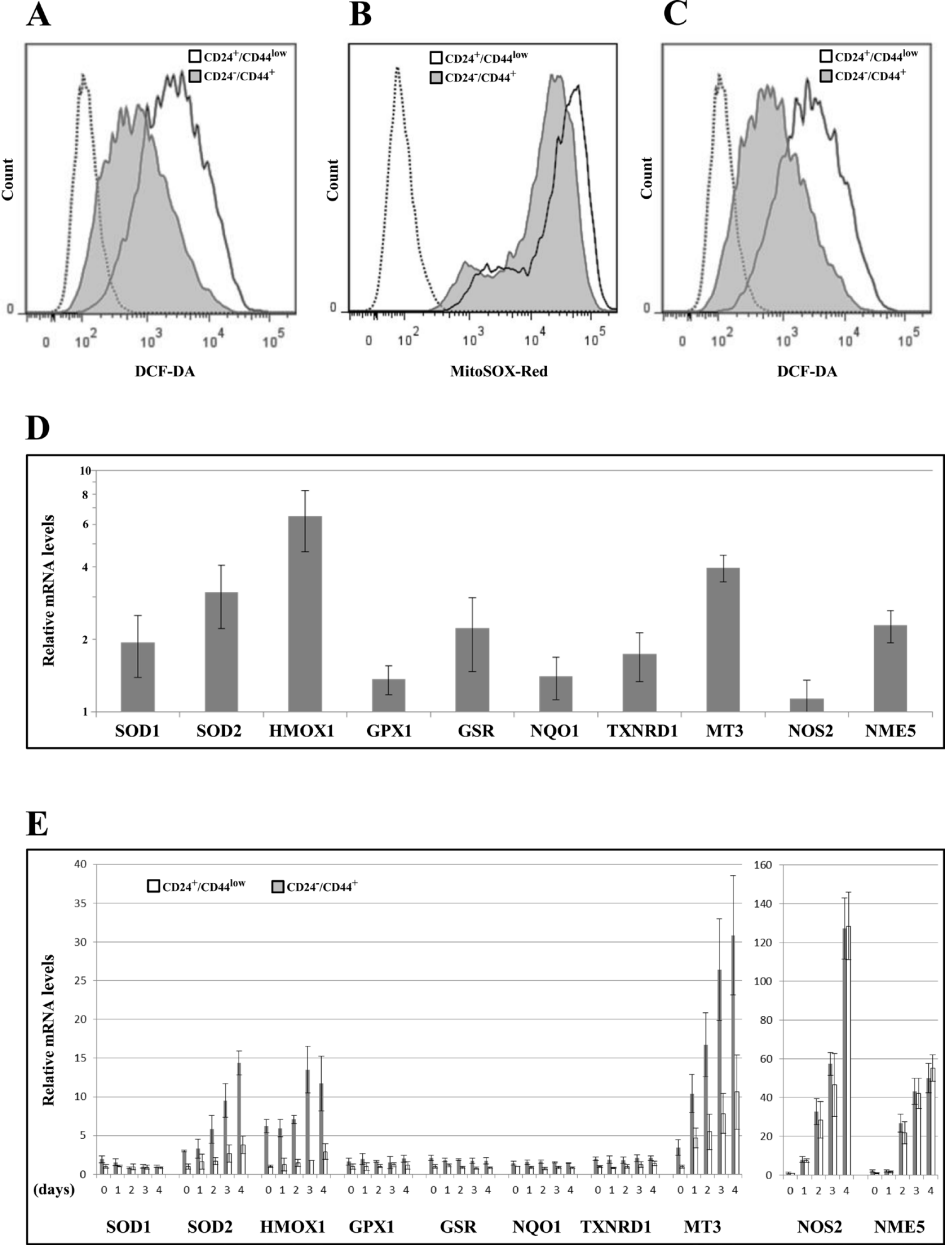
Analyses of ROS levels and expression of oxidative stress-related genes in CD24^+^/CD44^low^ cells and CD24^−/low^/CD44^+^ cells (**A**) Basal intracellular ROS concentrations were measured by DCF-DA staining, (---- negative control without DCF-DA probe). The flow cytometry analysis shown is representative of 3 independent experiments. (**B**) As in (A) but using MitoSOX red instead of DCF-DA (---- negative control without MitoSOX red probe). (**C**) As in (A) but 2 days after 10 Gy irradiation. (**D**) Analysis by qRT-PCR of the relative expression of the mRNAs encoding stress-related genes. Normalization was performed as indicated in Materials and Methods. For each gene, expression in CD24^+^/CD44^low^ cells was normalized to 1 and the ratio of relative mRNA level of CD24^−/low^/CD44^+^ cells to CD24^+^/CD44^low^ cells was presented. Each value corresponds to the mean value of at least 2 independent PCRs performed from 3 independent experiments. Error bars correspond to SEM. (**E**) Analysis by qRT-PCR of the relative expression of the mRNAs encoding stress-related genes during 4 days after 10 Gy irradiation. Normalization was performed as indicated in Materials and Methods. For each gene, expression at day 0 in CD24^+^/CD44^low^ cells was normalized to 1.

Low ROS levels and high ROS defenses have been ascribed [8], albeit not systematically [13], to the CSC phenotype in breast tumors. We thus studied whether changes in ROS levels characterize TGFβ-induced CD24^−/low^/CD44^+^ cells during EMT. First, we measured the intracellular concentrations of ROS prooxidants using 2′-7′-dichlorofluorescein diacetate (DCF-DA) staining. CD24^−/low^/CD44^+^ cells contained significantly lower concentrations of ROS than CD24^+^/CD44^low^ cells (Figure 5A and Supplementary Figure 4). Similar results were obtained using MitoSox-Red, a highly selective probe for mitochondrial superoxide (Figure 5B). Upon irradiation, ROS were maintained at lower levels in CD24^−/low^/CD44^+^ cells than in their parental counterparts (Figure 5C and Supplementary Figure 4), indicating that the differences in ROS levels also persisted during the mitotic blockade when cell death occurred.

We next determined whether ROS modulation during EMT could be related to differential regulation of oxidative stress-related genes. We studied the expression of 10 genes involved (directly or indirectly) in the control of oxidative stress balance, by RT-PCR before (Figure 5D) and during the 4 days following irradiation (Figure 5E). Notably, 9 of these genes were significantly upregulated in non-irradiated CD24^−/low^/CD44^+^ cells *versus* their CD24^+^/CD44^low^ counterpart cells (SOD1, SOD2, HMOX1, GSR, NQO1, TXNRD1, MT3, NME5), suggesting greater depletion of ROS. After irradiation, 5 of these genes were induced (SOD2, HMOX1, MT3, NOS2, NME5) in both populations and the first 3 of these genes were induced more in CD24^−/low^/CD44^+^ cells (Figure 5E and Supplementary Figure 5). Interestingly, we previously observed that MT3 expression is directly modulated by CD24 expression [20], suggesting a potential role of CD24 in the acquisition of antioxidant activity during TGFβ-induced EMT.

Taken together, these data indicate that EMT induced a complex deregulation of a set of actors involved in ROS metabolism, leading to high levels of antioxidant defense systems and low levels of intracellular ROS in CD24^−/low^/CD44^+^ cells. Furthermore, in response to ionizing radiation, radioresistant CD24^−/low^/CD44^+^ cells were able to upregulate, more efficiently than radiosensitive CD24^+^/CD44^low^ cells, the expression of a few ROS detoxication genes. Thus, because sustained low ROS levels contributed to tumor radiation resistance, ROS control appeared to be a causal key event in the acquisition of radioresistance during EMT.

### Death receptor pathways are less activated after irradiation in CD24^−/low^/CD44^+^ than in CD24^+^/CD44^low^ cells

**Figure 6:**
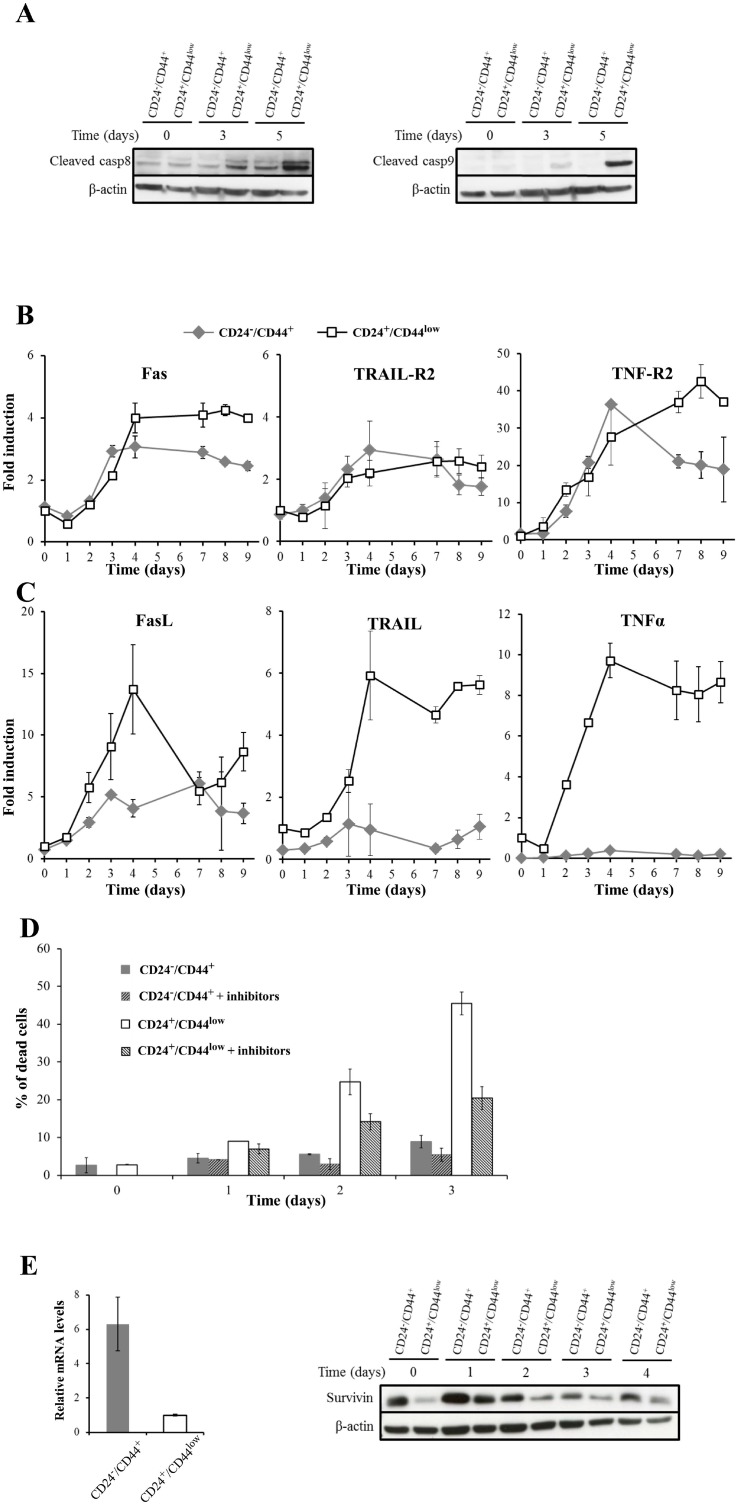
Role of death receptor pathways in radiation-induced cell death of CD24^+^/CD44^low^ cells and CD24^−/low^/CD44^+^ cells (**A**) Kinetics of cleavage of caspases 8 and 9 at different times after 10 Gy irradiation. The Western blots shown are representative of 3 independent experiments. (**B**) and (**C**) mRNA levels of death receptors and corresponding ligands after a 10 Gy irradiation of CD24^+^/CD44^low^ cells and CD24^−/low^/CD44^+^ cells. These mRNA levels were determined by quantitative RT-PCR. Normalization was performed as indicated in Materials and Methods and the basal expression of CD24^+^/CD44^low^ cells on day 0 was normalized to 1. Each value corresponds to the mean value of at least 2 independent PCRs performed from 3 independent experiments. Error bars correspond to standard deviation. (**D**) Neutralization of the death receptor pathways reduces radiation-induced apoptosis. CD24^+^/CD44^low^ cells and CD24^−/low^/CD44^+^ cells were 10 Gy-irradiated and treated or not with a combination of Fas/Fc, TRAIL-R1/Fc and TNF-R1/Fc chimera (250 ng/mL, 100 ng/mL and 100 ng/mL, respectively). These chimeras, which are cytokines designed to neutralize apoptosis induced by the corresponding ligand, were added to the culture medium at days 0 and 2. Viable and dead cells were counted after trypan blue staining. Results correspond to the mean ± standard deviation of 3 independent experiments. (**E**) Survivin expression by quantitative RT-PCR (left) and Western blot (right) after a 10 Gy irradiation of CD24^+^/CD44^low^ cells and CD24^−/low^/CD44^+^ cells. For RT-PCR, normalization was performed as indicated above, and the Western blot shown is representative of 3 independent experiments.

As we demonstrated that late after γ-irradiation, HMLE cells died of caspase-dependent apoptosis associated with mitotic catastrophe, the involvement of upstream apoptotic pathways in late cell death were explored. Caspase 7, like caspase 3, belongs to the executioner/effector proteases activated either by the extrinsic apoptotic pathway (involving death receptors and caspase 8) and/or by the intrinsic pathway (involving mitochondrial alterations and caspase 9). Starting 3 days after irradiation, caspase 8 and caspase 9 were activated by cleavage more efficiently in CD24^+^/CD44^low^ than in CD24^−/low^/CD44^+^ cells (Figure 6A). These results showed the involvement of the extrinsic apoptosis pathway and suggested either that the intrinsic pathway has been concomitantly activated or that HMLE cells are type II cells in which caspase 8 mediates the proteolytic cleavage of the BH3-interacting domain death agonist (BID), leading to the generation of a mitochondrion-permeabilizing fragment (tBID) and caspase 9 activation [21]. In a set of breast cancer cell lines, we previously showed that delayed apoptosis following irradiation was due to death receptor pathway activation [19]. We therefore investigated whether modulation of radiosensitivity after EMT could be due to differential activation of extrinsic apoptosis. Extrinsic apoptosis is related to activation of the 3 main death receptor pathways (FasL, TRAIL and TNF-α), hence mRNA levels of the main death receptors (Fas, TRAIL-R1 / TRAIL-R2 and TNF-R1 /TNF-R2) and their ligands (FasL, TRAIL and TNF-α, respectively) were measured before and after radiation (10 Gy) exposure. Expression of TRAIL-R1 and TNF-R1 did not differ significantly between the cell cultures and was not further modulated after irradiation (Supplementary Figure 6). In contrast, expression of Fas, TRAIL-R2 and TNF-R2 was significantly upregulated upon irradiation (Figure 6B): their overexpression started 2 or 3 days after irradiation and lasted for 9 days. Death receptor expression was similar in the two cell cultures during the first days following irradiation, but after few days slight but significant long-term overexpression of Fas and TNF-R2 was detected in CD24^+^/CD44^low^ cells (Figure 6B).

Such delayed overexpression was also observed for the 3 ligands, and these effects were much stronger in CD24^+^/CD44^low^ than in CD24^−/low^/CD44^+^ cells, with apparent differences in kinetics and persistence for each ligand (Figure 6C). FasL was induced and differentially expressed when comparing the 2 cell cultures during the first 4 days, and then the expression remained high but identical. TRAIL expression was induced and differentially expressed in both cultures for 9 days after irradiation. TNF-α was strongly overexpressed and remained at a persistently high level essentially in CD24^+^/CD44^low^ cells, while its expression remained low in CD24^−/low^/CD44^+^ cells. These results were confirmed at the protein level by FACS / ELISA analysis (Supplementary Figure 7 and 8).

To establish whether activation of death receptor pathways was a causal mechanism in radiation-induced apoptosis of HMLE cells, we used soluble recombinant chimera (Fas/Fc, TRAIL-R1/Fc and TNF-R1/Fc) to inhibit the apoptosis induced by the corresponding ligands (soluble as well as surface ligands). Both cell cultures were treated with the 3 inhibitors and irradiated (10 Gy), and cell death was analyzed for 3 days (Figure 6D). The concomitant addition of the 3 inhibitors significantly decreased the percentage of dead cells and diminished the slope of the percentage of dead cells over time.

Finally, members of the family of endogenous caspase inhibitors (IAPs) have been reported to counteract the function of apoptotic proteins, and survivin is specifically overexpressed and plays an important role in regulating the resistance of tumors, including breast carcinomas, to apoptosis [22, 23]. Supporting this notion, we observed that survivin mRNA level was substantially higher in CD24^−/low^/CD44^+^ than in CD24^+^/CD44^low^ cells before and after radiation exposure (Figure 5E), and, at the protein level, we found an inverse correlation between survivin and cleaved caspase 7 levels (Figure 6E). Because survivin can bind to caspases, including caspase 7, and inhibit their activation, differential survivin expression appears to be another level of control of differential activation of irradiation-induced apoptosis between CD24^−/low^/CD44^+^ and CD24^+^/CD44^low^ cells.

Altogether, these results indicate that activation of death receptor pathways mediates radiation-induced apoptosis in CD24^+^/CD44^low^ cells and that these pathways are mainly repressed at the transcriptional level in CD24^−/low^/CD44^+^ cells. This feature appears to be a causal key event in the intrinsic radioresistance of CD24^−/low^/CD44^+^ cells.

## DISCUSSION

Our data provide evidence that mammary CD24^−/low^/CD44^+^ CSCs generated during TGFβ-induced EMT are more radioresistant than parental CD24^+^/CD44^low^ cells. In the literature, radioresistance properties are frequently associated with CSCs, but investigations of the intrinsic radiosensitivity of CSCs have yielded controversial results. Various studies show a radioresistant phenotype of CSCs [6], while others conclude that there is no difference in the radiosensitivity of CSCs and non-CSCs, or even that CSCs are more sensitive [11–14]. There may be several sources of discrepancies in the literature: 1/ As expected, cellular models could be a source of variability. In breast CSC studies, results have been obtained by comparison of breast cancer cell lines with distinct tumorigenicity, the more aggressive supposedly being enriched in CSCs. In other studies, mesenchymal tumor cells with stem-like properties were obtained after ectopic expression of transcription factors (TFs) involved in the EMT/CSC phenotype or forced expression of H-RAS^G12V^, which also leads to EMT [1, 2], but whether the CD24^low^/CD44^high^ cells obtained were equivalent and had all the properties of “purified” mesenchymal CD24^low^/CD44^high^ cells remains unclear. 2/ Another source of discrepancies could be the markers used to define CSCs. The specificity of these markers remains controversial and the subpopulations detected by different combinations of markers only partially overlap [24]. Taken together, most studies have failed to provide clear indications of the radiosensitivity of mammary CSCs generated during EMT in situations mimicking tumor behavior.

In our study, the cellular model has the advantage of using “purified” CD24^+^/CD44^low^ and CD24^−/low^/CD44^+^ cells with the same genetic background. Therefore, modulation of the radiosensitivity of these cells could be exclusively ascribed to genetic reprogramming/dedifferentiation due to production of CD24^−/low^/CD44^+^ stem-like cells in the tumor through epigenetic changes induced by epithelial mesenchymal plasticity.

The radioresistant phenotype of CD24^−/low^/CD44^+^ cells was associated with increased clonogenic survival, as well reduced cell death rate after irradiation. As previously described, γ-irradiation of breast cancer cells led to delayed apoptosis after prolonged growth arrest in a context of mitotic catastrophe [18, 19]. Apoptosis started 2 days after irradiation and was sustained for at least 11 days in both cell cultures, although the rate of cell death in irradiated CD24^−/low^/CD44^+^ cells was always lower than in CD24^+^/CD44^low^ cells. Differential cell death led to an increased potential of generation of progeny for CD24^−/low^/CD44^+^ cells. Our results indicate that cell cycle distribution and free-radical scavengers, but not DNA repair, are intrinsic cellular determinants that drive radiosensitivity.

Differences in cell cycle distribution are essentially related to a an increased proportion of polyploid cells in CD24^+^/CD44^low^ cells, reflecting an increased level of genetic instability in these cells. The high level of polyploid cells reinforces the long-term persistence of cell death as well as the loss of clonogenic capacities of irradiated CD24^+^/CD44^low^ cells. However, the similarity in the time course of disappearance of γ-H2AX foci for both cell cultures indicates that such differential radiation-induced mitotic slippage leading to polyploidy is not related to differential overall DNA repair capacities.

ROS are known to mediate the effects of ionizing radiation [25], and low ROS levels and radiation resistance are frequently associated in CSCs, especially in breast CSCs [7, 8]. Twist1 and Twist2 EMT-related transcription factors are strongly overexpressed in CD24^−/low^/CD44^+^ cells in our model (Figure 1C) and have been reported to increase ROS scavengers and lower the level of intracellular ROS [26]. Accordingly, we clearly observed lower ROS levels in CD24^−/low^/CD44^+^ than in CD24^+^/CD44^low^ cells in basal non-irradiated conditions as well as in cells after irradiation. We show that a low ROS level in CD24^-^/CD44^+^ cells is strongly associated with overexpression of a set of genes directly or indirectly involved in the control of the Red-Ox balance. Our set of genes partially overlaps the group of ROS genes proposed by Diehn *et al.* [8] as a signature to discriminate CSCs from non-stem cells in human breast tumors. Among the 9 genes overexpressed in CD24^−/low^/CD44^+^ cells, 5 are induced by irradiation, and 3 (SOD2, HMOX1 and MT3) are differentially overexpressed in CD24^−/low^/CD44^+^ cells. So, differential regulation of radiation-induced ROS genes also appears to be involved in acquisition of radioresistance by CD24^−/low^/CD44^+^ cells. Interestingly, our group previously reported that forced extinction of CD24 promotes radiation resistance through the control of oxidative stress, and MT3 expression was directly downregulated by CD24, before and after irradiation [20]. Moreover, MT3 overexpression is correlated with a poor prognosis in breast cancer [27], plays a role in oxidative stress regulation [28] and inhibits proliferation of breast cancer cell lines [29]. So, MT3 regulation by CD24 suggests a potential role of this gene in proliferation, as well as in the low ROS levels in CD24^−/low^/CD44^+^ cells. An effort to decipher ROS gene function during the radiation response is currently underway.

Irradiated breast cancer cells die by apoptosis due to mitotic catastrophe [19, 30]. The extrinsic death receptor pathways drive the delayed cell death, and our results show for the first time that radioresistance of stem-like breast cancer cells could be attributed to a lower activation of these pathways after irradiation. We show induction of expression of the main members of the death receptors pathways, but the triggering of apoptosis is mediated by concomitant overexpression of the death receptors ligands: FasL, TRAIL and TNF-α. The observation that neutralization of the 3 pathways significantly reduces apoptosis clearly indicates that these pathways play a causal role and that late apoptosis results from the sum of the deregulation of these 3 pathways. The 3 ligands are differentially expressed in CD24^−/low^/CD44^+^ and CD24^+^/CD44^low^ cells, the level of expression remaining much lower in irradiated CD24^−/low^/CD44^+^ cells. The kinetics of expression of the members of these pathways show that the deregulation of expression induced by irradiation is maintained for at least 9 days, in agreement with the persistent radiation-induced cell death observed in Figure 2A. So, the persistence of differential expression of the death receptor ligands drives the persistent differential rate of cell death observed between the two cell cultures. The involvement of differential activation of death receptor pathways in acquisition of radioresistance is reinforced by the overexpression of survivin in CD24^−/low^/CD44^+^ cells. Expression of survivin is strongly related to loss of apoptosis in breast carcinomas [23], and treatment with survivin antisense significantly enhances TRAIL-induced apoptosis [31, 32]. Taken together, our results indicate that TGFβ-induced cell reprogramming of HMLE breast cells leads to the generation of radioresistant mesenchymal CD24^−/low^/CD44^+^ CSCs. The radioresistant cell phenotype generated by EMT through CSCs is associated with the activation/repression of opposing life-death signals. At the transcriptional level, pro-apoptotic death receptor pathways are inhibited, whereas anti-apoptotic survivin expression is increased.

In conclusion, our results clearly show that breast CD24^−/low^/CD44^+^ CSCs display an intrinsic radioresistant phenotype. Avoidance of mitotic slippage, polyploidy, mitotic catastrophe and free radical scavenger control appear to be crucial determinants of radiosensitivity, and transcriptional modulation of apoptotic death receptor pathways is essential for the control of irradiation-induced cell death. Our data demonstrating the radioresistance of breast CSCs provide additional evidence of the deleterious role of CSCs in aggressive tumor recurrence after radiotherapy.

## MATERIALS AND METHODS

### Cell culture

The human mammary epithelial HMLE cell line was kindly provided by RA Weinberg. HMLE cells have been derived from HMC and HME cells by sequential insertion of vectors coding for h-TERT and t/T antigens. As a consequence, HMLE cells are deficient for a functional p53 regardless the level of expression for CD24 or CD44. Cells were grown in adherent conditions, maintained in 1:1 Dulbecco's Modified Eagle Medium (DMEM) /HAMF12 medium supplemented with 10% fetal calf serum, penicillin, streptomycin, amphotericin B (antibiotic-antimycotic mix) (all from Life Technologies, Cergy-Pontoise, France) and 10 ng/mL human epidermal growth factor (EGF) (Sigma), 0.5 μg/mL hydrocortisone (Sigma) and 10 μg/mL insulin (Sigma). All cell cultures were done in 5% CO_2_ and 95% humidity. EMT was induced after prolonged treatment with 2.5 ng/mL recombinant TGFβ1 (Life Technologies, Cergy-Pontoise, France). Because of the short half-life of TGFβ, TGFβ -supplemented medium was changed every 48 hours. Cell proliferation and survival analyses were performed, 5–10 days after FACS sorting of CD24^+^/CD44^low^ and CD24^−/low^/CD44^+^ cells, in 2 or more independent experiments, by scoring at least 300 cells each time. Discrimination between viable and dead cells was performed by trypan blue exclusion.

### Chemical, reagents and antibodies

All biochemicals were from Sigma (Saint Quentin Fallavier, France) unless otherwise specified. Antibodies against CD24 (clone ML5), CD44 (clone C26), N-Cadherin (clone 8C11), TRAIL and isotypic controls were from BD Biosciences (San Jose, CA, USA). Antibodies against TNF-R1, TNF-R2 and soluble recombinant chimera (Fas/Fc, TRAIL-R1/Fc and TNF-R1/Fc) were from R&D Systems (France). Antibody against TRAIL-R1 (clone DIR1) was from Cliniscience (France). Antibody against TRAIL-R2 (clone DIR2-4) was from eBioscience (ThermoFisher scientific). Antibody against E-Cadherin (clone 67A4) was from Exbio (Czech Republic). Antibodies against FasL and Fas were from Santa Cruz Biotechnology (USA). Antibodies against survivin, cleaved caspase 3, 7, 8 and 9 were from Cell Signaling Technology (The Netherlands). For cell cycle analyses, propidium iodide or topro3 was used for DNA quantification and immuno-studies were performed with phospho-histone H3 (Ser10) (Cell Signaling #8552) and cyclin B1, C-ter, clone Y106, (Millipore # 04-220) and Goat anti-Rabbit Alexa Fluor 405 (ThermoFisher #A-31556 ) antibodies.

### Irradiation

For all experiments except clonogenic assays, cells were plated at least 24 h prior to irradiation. On day 0, cells were γ-irradiated using a ^137^Cs irradiation unit at a dose rate of ~2 Gy/min, and then incubated with fresh medium. In every experiment, studied cells were 10 Gy-irradiated and control cells were submitted to sham irradiation. For clonogenic assays cells were trypsinized and immediately irradiated in suspension for the time required to generate a dose curve of 0, 2, and 4 Gy.

### Clonogenic assays

Colony-forming assays were performed immediately after irradiation by plating cells into 6-well plates, in triplicate. Fixation was performed 7 days after plating. Cells were fixed for 30 min in 4% paraformaldehyde and washed and stained overnight in methylene blue/30% methanol. Colonies containing more than 50 cells were counted. The surviving fraction for each radiation dose was normalized to that of the non-irradiated sample, and points were fitted using an exponential tendency curve. At least 3 independent experiments were performed.

### Cell staining

Cancer stem cell marker labeling and analysis were performed as described in Bensimon *et al*.[18, 20]. For cell cycle analysis, cells were fixed in 70% ethanol overnight, and stained with 0.025 mg/mL propidium iodide. For ROS analysis, after soft trypsinization cells were loaded with 2 mM DCFH2-DA (Invitrogen) and incubated for 30 min at 37° C. Labeled cells were washed, refrigerated on ice and immediately analyzed by flow cytometry.

### Flow cytometry

Cells were analyzed on a SORP LSR-II analyzer (Configuration: 488 nm, 561 nm, 405 nm, 355 nm, and 635 nm) or on a BD FACSCalibur (Configuration: 488 nm and 635 nm) (BD Biosciences, San Jose, CA). Data were analyzed with FlowJo v7.6.1 (Tree Star). Cells were sorted on a BD Influx sorter (BD Biosciences) (Configuration: 488 nm, 561 nm, 405 nm, 355 nm, and 635 nm). For each experiments, 10000 to 20000 cells were analyzed.

### Immunohistochemistry

γ-H2Ax foci were analyzed as previously described [33].

### RNA extraction and quantitative real-time PCR

Total RNA was extracted from frozen cell pellets with a phenol/chloroform/isopropanol protocol, using RNA Instapure reagent (Eurogentech, Belgium). cDNA synthesis was performed with the SuperScript VILO^®^ cDNA Synthesis Kit (Life Technologies, Cergy-Pontoise, France) according to the manufacturer's recommendations. RT-PCR was performed with an ABI Prism 7300 detection apparatus (Applied Biosystems, Courtaboeuf, France) using the Taqman Universal Master Mix according to the manufacturer's recommendations. The Ct value was determined with the Sequence Detection System software. All the primers were from Applied Biosystems. Levels of gene expression were determined using GENORM software and normalized using GAPDH and RPLPO.

### Western blot

Cell lysates were prepared by cell sonication in a buffer containing 10 mM Hepes pH7.5, 5 mM KCl, 1.5 mM MgCl2, 2.5 mM EDTA, 0.5 mM DTT, 2.5 mM PMSF, 5 mM iodoacetamide, 0.5% NP-40 and protease inhibitor cocktail (CompleteTM, Roche). Cell lysates (10 μg) were subjected to sodium dodecyl sulfate–polyacrylamide gel electrophoresis and proteins were transferred and detected using a chemiluminescence procedure.

## SUPPLEMENTARY MATERIALS AND FIGURES


