# Supplementary material for: Breast cancer stem cell-like cells generated during TGFβ-induced EMT are radioresistant
Source: Oncotarget. 2018 May 4;9(34):23519–31. doi: 10.18632/oncotarget.25240 (PMC5955125; doi:10.18632/oncotarget.25240)
Supplement: Supplementary file 1 [file oncotarget-09-23519-s001.pdf]

Breast cancer stem cell-like cells generated during TGFβ-induced EMT are radioresistant

SUPPLEMENTARY MATERIALS

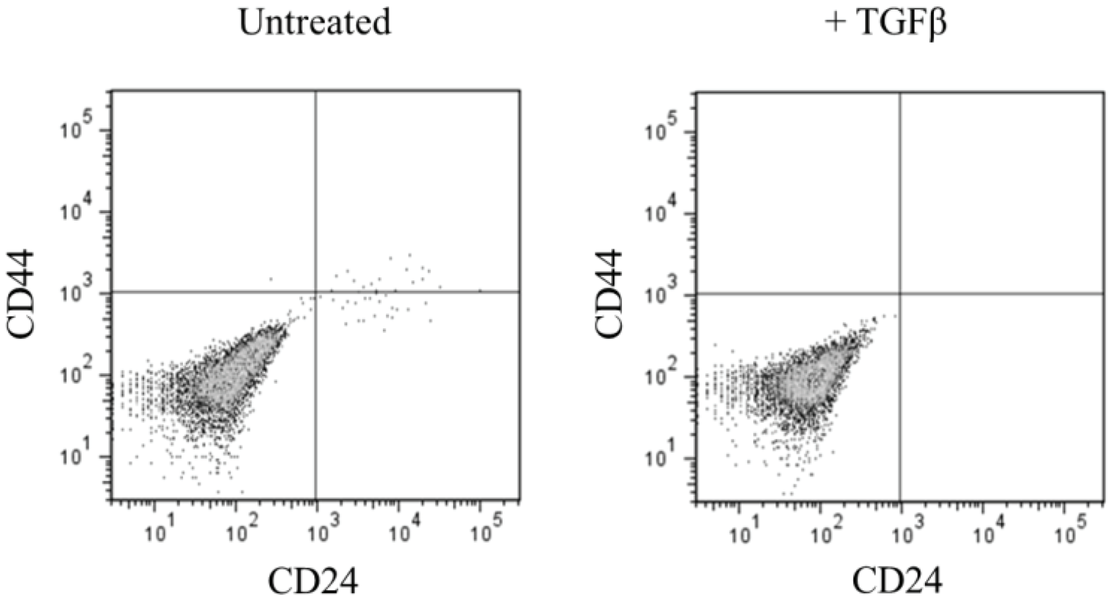

Supplementary Figure 1: Isotypic controls for Figure 1B in which CD24 and CD44 labellings of HMLE cells is shown for parental cells (left) and TGFβ-induced cells (right).

	$CD24^{-/low}/CD44^{+}$	$CD24^{+}/CD44^{low}$
0 Gy	1 ± 0.11	1.26 ± 0.23
10 Gy (day 4)	1.70 ± 0.31	5.01 ± 1.25

Supplementary Figure 2: Relative mean fluorescence intensities of the FACS analyses presented in Figure 2C for the cleaved caspase 3. Mean fluorescence intensity detected in control $CD24^{-/low}/CD44^{+}$ cells were used for the normalization (to 1). Results correspond to the mean ± SD of 3 independent experiments.

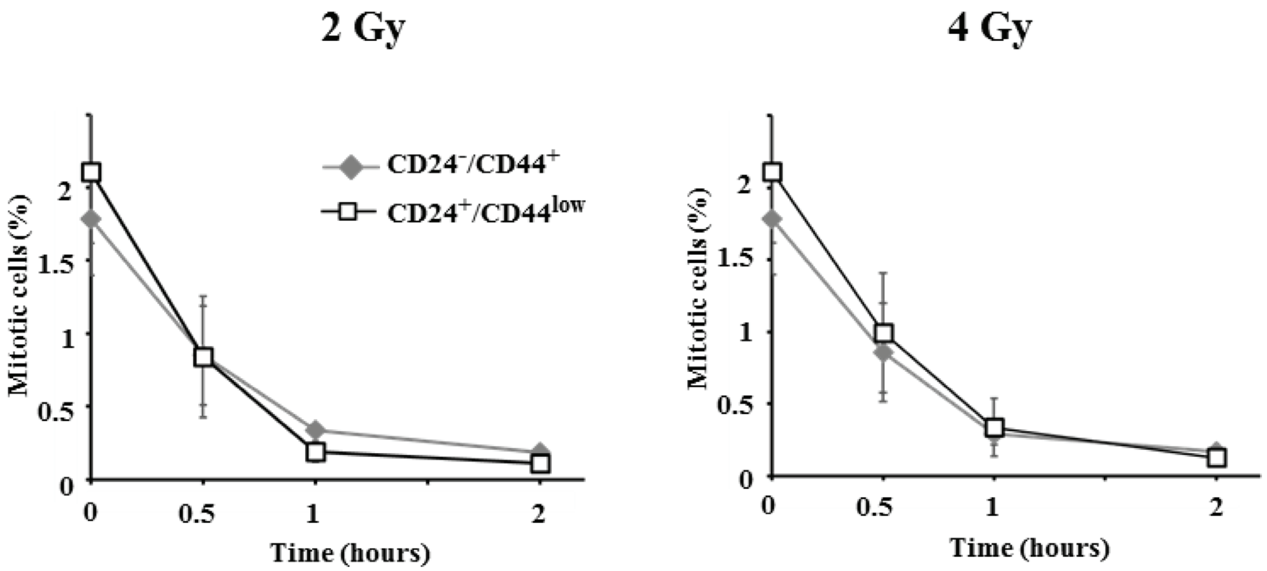

Supplementary Figure 3: Initial time course of the mitotic index of CD24^{+}/CD44^{low} and CD24$^{–/low}$/CD44^{+} cells upon irradiation. The mitotic indexes were determined by phospho-histone H3 labelling in independent experiments (*n* = 2 for the 2 Gy and *n* = 3 for the 4 Gy series) and resulting means and standard deviations were plotted.

	CD24$^{-/low}$/CD44^{+}	CD24^{+}/CD44^{low}
0 Gy	1 ± 0.23	7.02 ± 0.83
10 Gy (day 2)	1.12 ± 0.18	7.95 ± 1.85

Supplementary Figure 4: Relative mean fluorescence intensities corresponding to DCF-DA FACS analyses presented in Figure 5A and 5C. Mean fluorescence intensities detected in CD24$^{–/low}$/CD44^{+} cells were normalized to 1. Results correspond to the mean ± SD of 3 independent experiments.

		Day				
		0	1	2	3	4
SOD1	CD24$^{-/low}$/CD44^{+}	1.93 +/- 0.41	1.55 +/- 0.44	0.86 +/- 0.10	1.01 +/- 0.22	1.02 +/- 0.13
	CD24^{+}/CD44^{low}	1 +/- 0.22	1.04 +/- 0.11	0.94 +/- 0.35	0.91 +/- 0.24	0.84 +/- 0.12
SOD2	CD24$^{-/low}$/CD44^{+}	3.02 +/- 0.12	3.29 +/- 1.21	5.80 +/- 1.79	9.52 +/- 2.13	14.36 +/- 1.57
	CD24^{+}/CD44^{low}	1 +/- 0.12	1.61 +/- 0.95	1.71 +/- 0.44	2.67 +/- 1.09	3.79 +/- 1.09
HMOX1	CD24$^{-/low}$/CD44^{+}	6.21 +/- 0.85	5.94 +/- 1.11	7.07 +/- 0.54	13.52 +/- 3.02	11.72 +/- 3.56
	CD24^{+}/CD44^{low}	1 +/- 0.16	1.25 +/- 0.84	1.50 +/- 0.45	1.79 +/- 0.02	2.92 +/- 1.02
GPX1	CD24$^{-/low}$/CD44^{+}	1.65 +/- 0.45	1.96 +/- 0.68	1.65 +/- 0.11	1.52 +/- 0.76	2.00 +/- 0.47
	CD24^{+}/CD44^{low}	1 +/- 0.33	1.01 +/- 0.49	1.06 +/- 0.21	1.22 +/- 0.26	1.14 +/- 0.50
GSR	CD24$^{-/low}$/CD44^{+}	2.10 +/- 0.32	1.84 +/- 0.25	1.88 +/- 0.10	1.76 +/- 0.34	1.73 +/- 0.38
	CD24^{+}/CD44^{low}	1 +/- 0.22	1.15 +/- 0.17	0.88 +/- 0.17	0.80 +/- 0.16	0.86 +/- 0.11
NQO1	CD24$^{-/low}$/CD44^{+}	1.43 +/- 0.29	1.58 +/- 0.30	1.64 +/- 0.23	1.54 +/- 0.05	1.43 +/- 0.03
	CD24^{+}/CD44^{low}	1 +/- 0.00	0.87 +/- 0.13	0.73 +/- 0.16	0.88 +/- 0.14	0.85 +/- 0.13
TXNRD1	CD24$^{-/low}$/CD44^{+}	1.98 +/- 0.21	1.88 +/- 0.51	1.79 +/-0.42	2.08 +/- 0.46	2.07 +/- 0.27
	CD24^{+}/CD44^{low}	1 +/- 0.13	0.81 +/ 0.08	1.05 +/- 0.23	1.22 +/- 0.38	1.40 +/- 0.28
MT3	CD24$^{-/low}$/CD44^{+}	3.45 +/- 1.01	10.39 +/-2.47	16.72 +/- 4.16	26.41 +/- 6.58	30.84 +/- 7.70
	CD24^{+}/CD44^{low}	1 +/- 0.21	4.69 +/- 1.26	5.47 +/- 2.27	7.81 +/- 2.57	10.61 +/- 4.79
NOS2	CD24$^{-/low}$/CD44^{+}	1.20 +/- 0.46	8.03 +/- 1.50	32.66 +/- 6.62	57.30 +/- 5.91	127.17 +/- 15.85
	CD24^{+}/CD44^{low}	1 +/- 0.05	7.55 +/- 0.90	28.50 +/- 9.35	46.43 +/- 16.22	128.37 +/- 17.45
NME5	CD24$^{-/low}$/CD44^{+}	2.20 +/- 0.56	2.13 +/- 0.50	26.69 +/- 4.70	43.15 +/- 6.65	49.92 +/- 7.57
	CD24^{+}/CD44^{low}	1 +/- 0.35	1.71 +/- 0.52	21.81 +/- 5.60	42.13 +/- 7.74	55.24 +/- 6.89

Supplementary Figure 5: Quantification of the expression, measured by RT-PCR, for 10 genes involved in the control of oxidative stress balance, before and during the 4 days following irradiation (numerical data for Figure 5E). Normalization was performed as indicated in materials and methods. For each gene, expression at day 0 in CD24^{+}/CD44^{low} cells was normalized to 1.

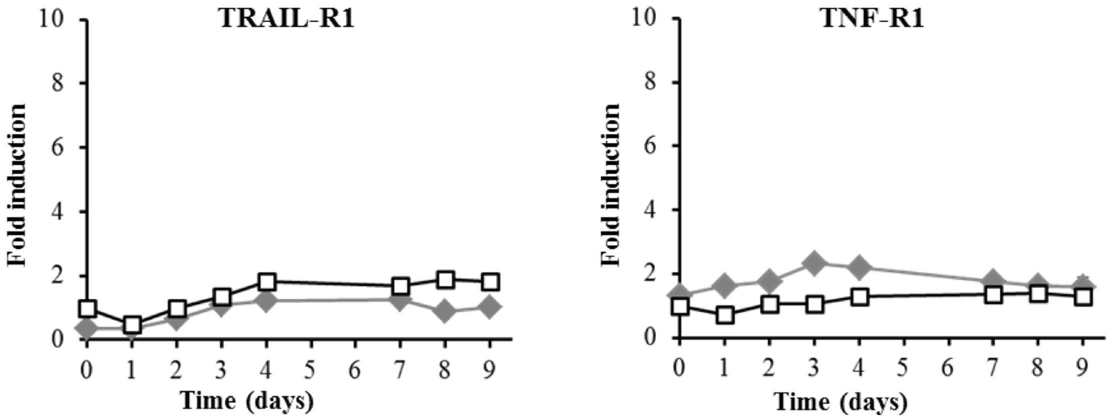

Supplementary Figure 6: mRNA levels of TRAIL-R1 and TNF-R1 death receptors after a 10 Gy irradiation of CD24^{+}/CD44^{low} and CD24$^{-/low}$/CD44^{+} cells.

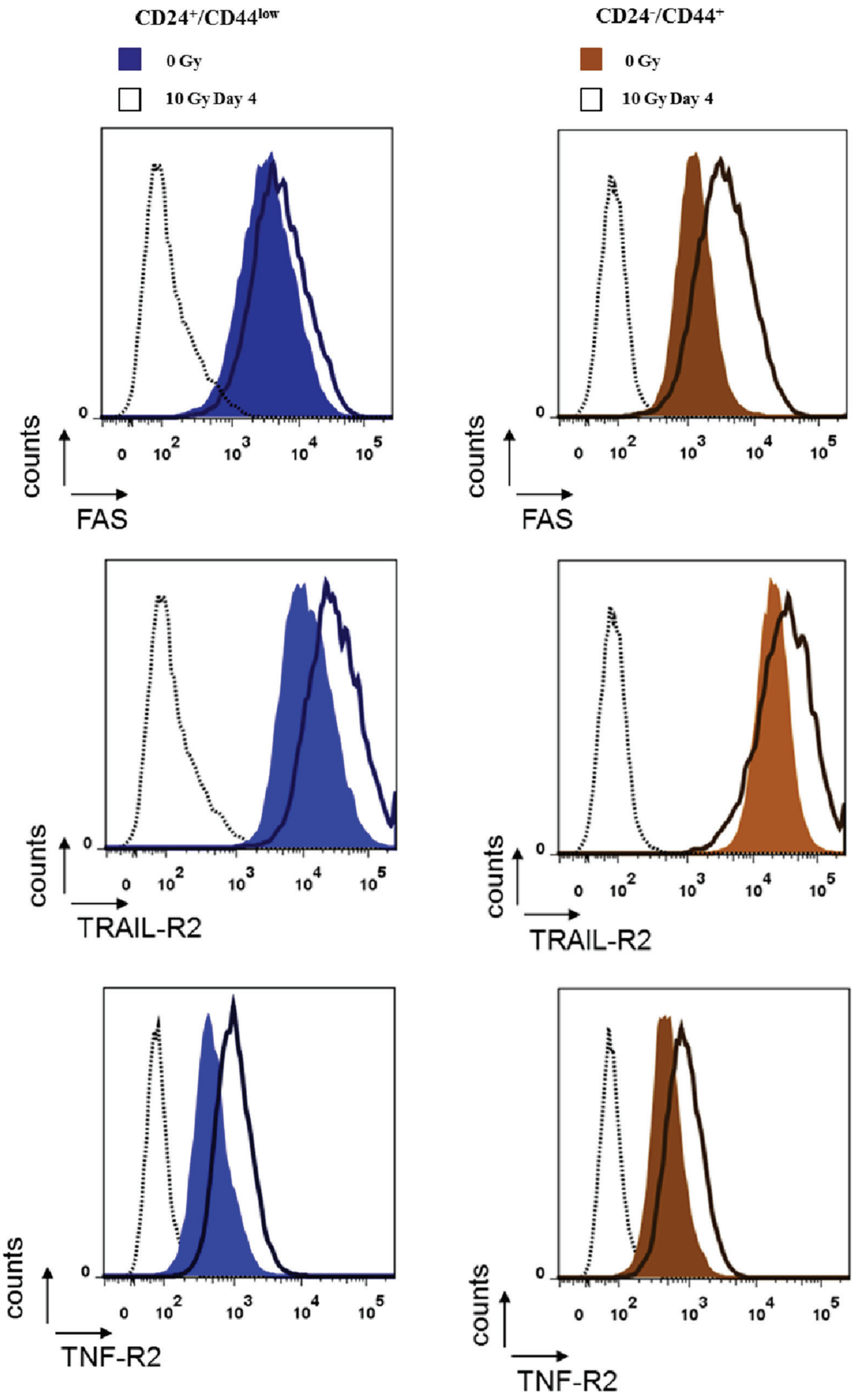

CD24+/CD44low
0 Gy
10 Gy Day 4
CD24-/CD44+
0 Gy
10 Gy Day 4
counts
FAS
counts
FAS
counts
TRAIL-R2
counts
TRAIL-R2
counts
TNF-R2
counts
TNF-R2
0
0
10^2
10^3
10^4
10^5

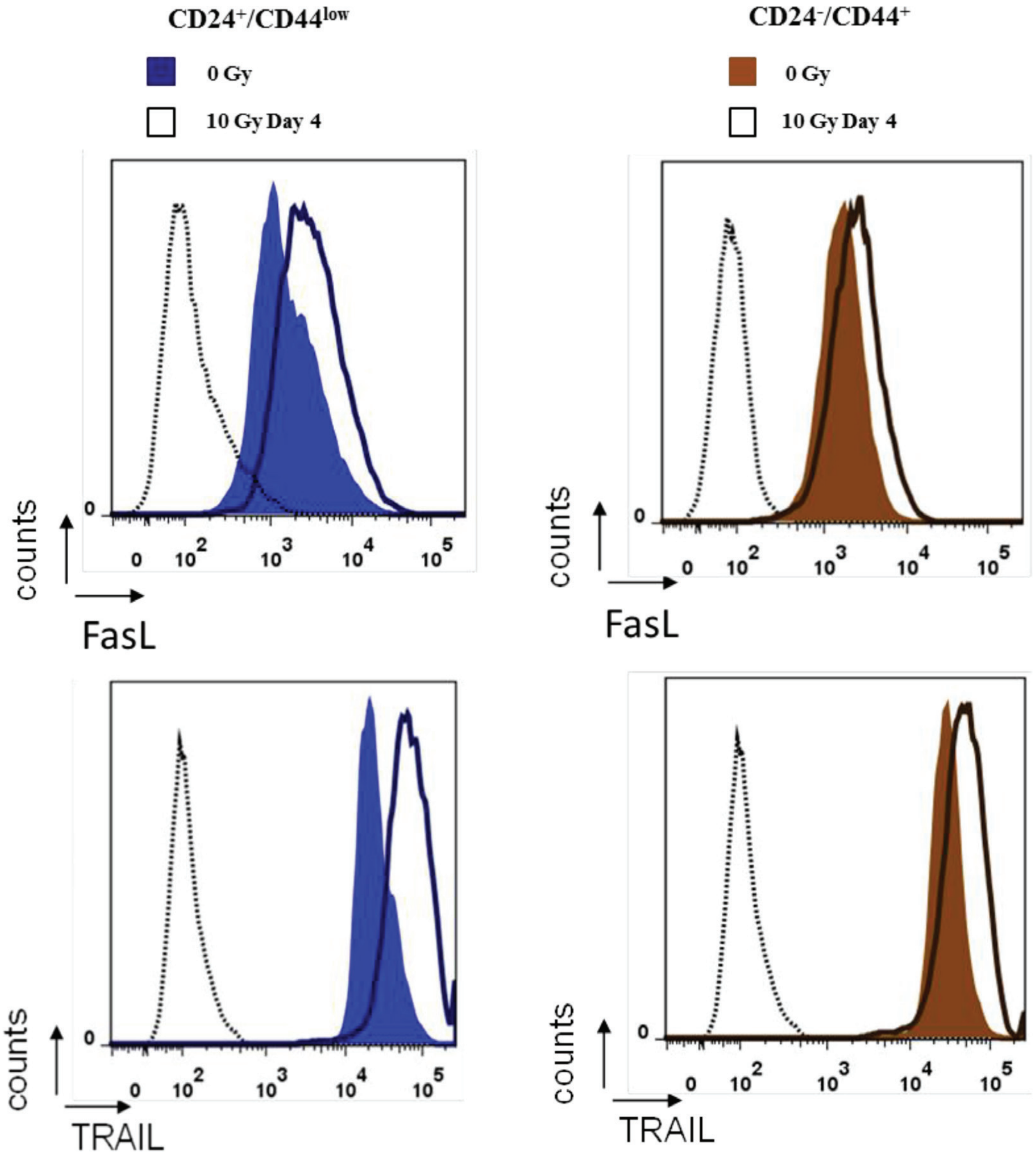

Supplementary Figure 7: Flow cytometry characterization of the expression of Fas, TRAIL-R2, TNF-R2 death receptors and FasL, TRAIL ligands, 4 days after a 10 Gy irradiation of CD24$^+$/CD44^{low} and CD24$^{-/low}$/CD44$^+$ cells. Similar results were obtained in 3 independent experiments.

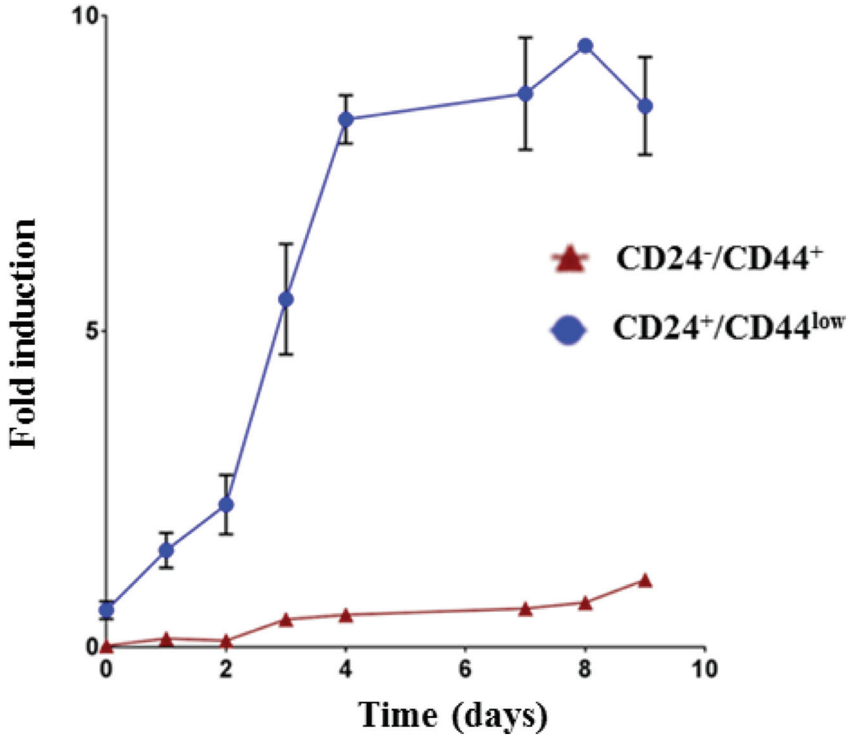

Supplementary Figure 8: Analysis of TNF-α secretion in culture medium of 10 Gy-irradiated $CD24^+/CD44^{low}$ and $CD24^{–/low}$/ $CD44^+$ cells. TNF-α was quantified daily by ELISA in supernatant of irradiated and non-irradiated cell cultures. Each value corresponds to the mean of at least 2 independent measurements from 2 independent experiments. Error bars correspond to standard deviation.
